# *Pediococcus acidilactici* Promotes the Longevity of *C. elegans* by Regulating the Insulin/IGF-1 and JNK/MAPK Signaling, Fat Accumulation and Chloride Ion

**DOI:** 10.3389/fnut.2022.821685

**Published:** 2022-04-01

**Authors:** Rui Hu, Yong Zhang, Weiyi Qian, Yan Leng, Yan Long, Xinjie Liu, Jinping Li, Xiangyuan Wan, Xun Wei

**Affiliations:** ^1^Zhongzhi International Institute of Agricultural Biosciences, Shunde Graduate School, Research Center of Biology and Agriculture, University of Science and Technology Beijing (USTB), Beijing, China; ^2^Beijing Beike Institute of Precision Medicine and Health Technology, Beijing, China; ^3^Beijing Engineering Laboratory of Main Crop Bio-Tech Breeding, Beijing International Science and Technology Cooperation Base of Bio-Tech Breeding, Beijing Solidwill Sci-Tech Co., Ltd., Beijing, China

**Keywords:** longevity, *C. elegans*, probiotic, *Pediococcus acidilactici*, chloride ion related genes

## Abstract

Probiotics are known to contribute to the anti-oxidation, immunoregulation, and aging delay. Here, we investigated the extension of lifespan by fermented pickles-origin *Pediococcus acidilactici* (PA) in *Caenorhabditis elegans (C. elegans)*, and found that PA promoted a significantly extended longevity of wild-type *C. elegans*. The further results revealed that PA regulated the longevity *via* promoting the insulin/IGF-1 signaling, JNK/MAPK signaling but not TOR signaling in *C. elegans*, and that PA reduced the reactive oxygen species (ROS) levels and modulated expression of genes involved in fatty acids uptake and lipolysis, thus reducing the fat accumulation in *C. elegans*. Moreover, this study identified the *nrfl-1* as the key regulator of the PA-mediated longevity, and the *nrfl-1/daf-18* signaling might be activated. Further, we highlighted the roles of one chloride ion exchanger gene *sulp-6* in the survival of *C. elegans* and other two chloride ion channel genes *clh-1* and *clh-4* in the prolonged lifespan by PA-feeding through the modulating expression of genes involved in inflammation. Therefore, these findings reveal the detailed and novel molecular mechanisms on the longevity of *C. elegans* promoted by PA.

## Introduction

Population aging has been a global problem, leading to a growing of longer healthy life expectancy (1). There will be huge numbers of elderly people (65 years old and above), which is over 20% of the total population across world at 2050 (2). Some countries are facing a challenge of rapid population aging, such as South Korea, Japan, and China. Aging and longevity are multifactorial complex processes, involving in both genetic and environmental factors (3).

Gut microbiota is the largest internal microenvironment in human body to communicate with the external world and its composition is shaped predominantly by environmental factors (4). In addition, microbiota acts as a mediator between host genes and environmental factors (5). Gut microbiota, with a 10-fold cell number and a 100-fold gene number greater than those of human, has been accepted as a potential marker in aging (6). Gut microbes that influence the immune system and metabolic process may shape the energy harvest, endow defense to pathogens, reduce oxidative stress, confer abilities to alleviate stress, and improve anti-inflammatory effect in anti-aging (7, 8). Probiotics, mainly *Lactobacillus* and *Bifidobacterium* from *lactic acid bacteria* (LAB) which are widely recognized beneficial bacteria for a long time and have been proved to increase the longevity of *Caenorhabditis elegans* (*C. elegans*) (9–12).

*C. elegans* is an ideal model for the longevity research of host-bacteria interaction due to a short life span, fast reproducible life, and using bacteria as food (13). Many longevity-associated signaling pathways, such as the insulin/insulin-like growth factor-1 (IGF-1) signaling, NSY-1/PMK-1 MAP kinase signaling, and Target of Rapamycin (TOR) signaling were found in *C. elegans* (14, 15). In recent years, new signaling pathways have been found, such as ATFS-1/unfolded protein response (UPRmt) and HLH-30/TFEB-autophagy (16, 17). However, the mechanism of gut microbes regulating longevity is not totally understood and there are other longevity associated signaling pathways yet to be investigated.

*Pediococcus acidilactici* (PA), also a member of LAB, was reported to regulate gut microbiota, reduce inflammation, release bacteriocin, and inhibit pathogenic bacteria in animals (18–20). PA is a strain isolated from naturally fermented pickles in Chongqing, China. In this study, we examined whether PA can extend the lifespan of *C. elegans* and explored the mechanism of its anti-aging effect.

## Materials and Methods

### Bacterial Strains and *Caenorhabditis elegans* Strains

*Escherichia coli* strain OP50 was provided by the Institute of Biophysics of Chinese Academy of Sciences and grown in Luria-Bertani (LB) broth (Oxoid, United Kingdom) at 37°C for 18 h. PA was isolated from fermented pickles and grown in de Man, Rogosa, and Sharpe (MRS) broth (Oxoid, United Kingdom) at 37°C for 18 h.

Wild-type N2, CB1370 [*daf-2 (e1370)* II], VC1167 [*sulp-6(ok1586)*], RB1139 [*clh-4(ok1162)*], XA900 [*clh-1(qa900)*], VC1795 [*nrfl-1*(*ok2292*)], VC222 [*raga-1 (ok386)* II], GR1307 [*daf-16 (mgDf50)* I], QQ202 [*daf-2(cv20[daf-2:gfp])*III], TJ356 [*zIs356 (daf-16:GFP)* IV], and VC8 [*jnk-1(gk7)*IV] were purchased from Caenorhabditis Genetics Center (CGC, University of Minnesota, Twin Cities, United States). All *C. elegans* strains were cultured at 20°C on the Nematode Growth Medium (NGM) plates using living *E. coli* strain OP50 as food.

### Longevity Assays in *Caenorhabditis elegans*

To measure the lifespan of *C. elegans* accurately, age-synchronized worms of the wild-type strain (N2) were grown on NGM agar plates containing live *E. coli* OP50 at 20°C, until they reached the young adult stage. Approximately 80–100 age-synchronized worms were then transferred to fresh NGM plates covered with lawns of *E. coli* OP50 or PA (21). The plates were incubated at 20°C, the worms were transferred every other day thereafter. The worms that showed no reaction to gentle stimulation were scored as dead, whereas the animals that crawled off the plate, exploded, or bagged were censored, the surviving nematodes were transferred to a fresh plate, and the numbers of live and dead worms were scored. Two independent lifespan assays were performed.

### Measurement of Body Size

For the body length measurement, approximately 20 wild-type L4 larvae were randomly selected and inoculated to bacterial NGM plates covered with the lawns of *E. coli* OP50 or PA. From the first day that *C. elegans* were transferred to fresh bacteria spreading NGM plates, the sizes of live worms were examined every other day until reaching 5 days of age, according to the previous research (22). The worms were visualized with a microscope (Olympus SZ61) using a 10× objective lens of a bright-field microscope. The area of worm’s projection was analyzed as the body size using the Olympus view software.

### Brood Size Measurement

The L4-stage worms were placed on bacterial NGM plates covered with the lawns of *E. coli* OP50 or PA and incubated at 20°C. These worms were transferred to fresh bacterial NGM plates every 24 h until the end of ovulation. The progeny was counted each day. This assay was performed for six consecutive days, which was the reproductive period of the worms. The sum of the plate algebra was taken as the total sub algebra of the worm. Brood size evaluation of each bacterial species contained twenty worms, and one worm was detected on one plate.

### Assessment of Pharyngeal Pumping Rate

The N2 worms were used to evaluate the age-related decline in muscle function by pharyngeal pumping rate measurement at room temperature (10). L4-stage worms were transferred to NGM plates covered with the lawns of *E. coli* OP50 or PA and incubated at 20°C. The pharyngeal pumping rates were analyzed on days 3, 6, and 9 by counting the pumping frequency of the terminal pharyngeal bulb of each worm within 60 s. Three independent trials were performed with ten worms for each bacterial species.

### Measurement of Intracellular Reactive Oxygen Species Level

Dichlorofluorescin diacetate (H_2_DCF-DA, Sigma) was used to determine the intracellular reactive oxygen species (ROS) concentration in N2 nematodes. L4-stage worms were treated with *E. coli* OP50 or PA for 4 days, and collected with M9 buffer. Bacteria were removed by washing three times with M9 buffer. Approximately 35 nematodes were transferred to a transparent-bottom black 96-well plate (Corning, United States). The plate containing 100 μl of H_2_DCF-DA at 100 mM diluted with phosphate buffer (pH 7.2). The fluorescence intensity was measured by a fluorescence microplate reader (Molecular Devices SpectraMax250, United States) at 485/535 nm, and samples were measured every 20 min for a total of 160 min (23).

### DAF-16 Localization and DAF-2 Fluorescence Assay

The strain TJ356 (with DAF-16:GFP) was used to detect the intracellular DAF-16 localization. Then, 3-day-old adult worms (L4 stage) were transferred to NGM plates covered with the lawns of PA or *E. coli* OP50. The worms were placed for 5 days at 20°C, collected and washed three times by M9 buffer. The worms were fixed on the slide containing 2% agarose, anaesthetized with 10 mM imidazole (Sigma), and covered with coverslips. The worms were photographed by using a green excitation channel of confocal scanning laser microscope. Images were analyzed using the Image J 8.4 software (24). The percentage of daf-16 localization (cytosolic, nuclear, and both) was calculated according to a recent report (25). The same method was used for the detection of DAF-2 fluorescence from QQ202 strain.

### Oil Red O Staining

The L4-stage worms were placed on NGM plates covered with the lawns of PA or *E. coli* OP50. The plates were incubated at 25°C for 4 days. The worms were collected with phosphate buffer saline (PBS, pH 7.2) plus with 1% Triton to avoid the adhesion of the worms and reduce the loss of the worms during operation. Paraformaldehyde solution (4%, w/v) was used for fixation (4°C, 15 min). Then, the worms were subjected to freeze-thaw cycles two times between a −80°C freezing for 20 min and 25°C water thawing for 5 min (26). In this way, the cuticle of the worms could be broken and the worms were fully stained. Discarding the supernatant after centrifuging, the worms were washed once by M9 buffer. Then, the worms were dehydrated in 60% isopropanol for 15 min in the shaker at the maximum speed. Subsequently, the worms were stained by incubation with 1 ml Oil Red O stain (Beyotime, China) for 4–6 h. Stained worms were washed to remove the dyestuff, fixed on a slide containing 2% agarose, and visualized with Leica IIC50W microscope. The image J 8.4 software was used to determine the lipid content of staining worms. At least three independent determinations were repeated.

### BODIPY C12 Uptake Assay

C1-BODIPY-C12 (Molecular Probes, Invitrogen) was dissolved in dimethyl sulfoxide (DMSO, Sigma) to obtain 5 mM stock solution. Then, the stock solution was diluted to 1 mM with PBS. Furthermore, 0.5 ml diluted solution was loaded to the surface of empty NGM plates. The worms were quickly transferred to the plate and placed at 20°C for 6 h (27). Finally, after washing three times with M9 buffer, the worms were visualized using the green excitation channel of confocal scanning laser microscope TCS SP8 (Leica, Wetzlar, Germany). Fluorescence intensity was measured using the image J 8.4 software.

### RNA Isolation and Quantitative Real-Time PCR (RT-qPCR) Analysis

Following an 8-day treatment with PA or *E. coli* OP50, the worms were washed with M9 buffer for three times, *n* = 200. Total RNA was extracted using TRIzol reagent (TIANGEN Biotech Co., Ltd., Beijing, China), and quantified with a NanoDrop 2000 spectrophotometer (Thermo Scientific, United States). The cDNA was synthesized by reverse transcription using the 5× All-In-One RT MasterMix (Applied Biological Materials Inc., G490, BC, Canada) according to the manufacturer’s instructions. Software Primer premier 6.0 was used to design oligonucleotides for quantitative real-time PCR (RT-qPCR) (Supplementary Table 1). The RT-qPCR was performed with TB Green^®^ Premix Ex Taq™ (TAKARA Inc., Dalian, China) and QuantStudio 5 Real-Time PCR Systems (Applied Biosystems) (28). Three independent experiments were performed, the relative gene expression was determined using the 2^–ΔΔCT^ method.

### Statistical Analysis

Statistical analysis was presented using unpaired Student’s *t*-test and one-way ANOVA was compared in multiple groups using GraphPad Prism version 8.0 software (GraphPad Software, San Diego, CA, United States). Data were expressed as means ± SD. The log rank test was used to test the significance of life span in different experimental treatment groups. Compared with the control group, statistical differences at the *p* < 0.05 or *p* < 0.01 level were defined to be significant.

## Results

### Evaluation of the Lifespan Prolongation of *Caenorhabditis elegans* Mediated by *Pediococcus acidilactici*

**FIGURE 1:**
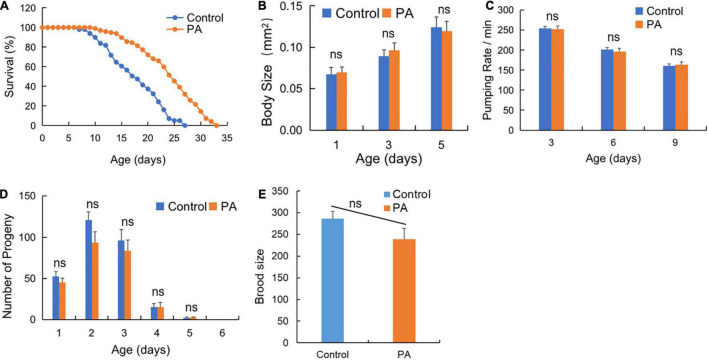
*Pediococcus acidilactici* (PA) extends lifespan but not affects basic physiology of wild-type *Caenorhabditis elegans (C. elegans)*. **(A)** Comparison of PA and *Escherichia coli (E. coli)* OP50 on the survival of *C. elegans*, *n* = 100. **(B)** The effects of PA on body size of *C. elegans*, *n* = 20. **(C)** The effects of PA on the pumping rate of *C. elegans*. **(D)** The effects of PA on the reproductive capacity of *C. elegans*, *n* = 20. **(E)** The effects of PA on brood size of *C. elegans*. ns, non-significant.

Recent advances in gut microbiota are rapidly propagating into the aging targets discovery and anti-aging probiotics screening practices (29). As the common probiotics, *Lactobacillus* and *Bifidobacterium* are well established for life extension research in *C. elegans* (30). However, PA has been mainly focused on its antimicrobial effects and bacteriocin production, while the anti-aging effects of PA are largely unknown. As shown in Figure 1A, the prolongation of *C. elegans* lifespan in the probiotic PA group was significantly higher than that in the control group (*p* < 0.001) fed by *E. coli* OP50. Moreover, we observed that PA-feeding had no significant influence on the basic physiology traits of the worms, such as body size, pumping rates, reproductive performance (progeny number), and brood size (Figures 1B–E).

### *daf-16*-Dependent Signaling Pathway Contributes to the *Pediococcus acidilactici*-Mediated Longevity of *Caenorhabditis elegans*

**FIGURE 2:**
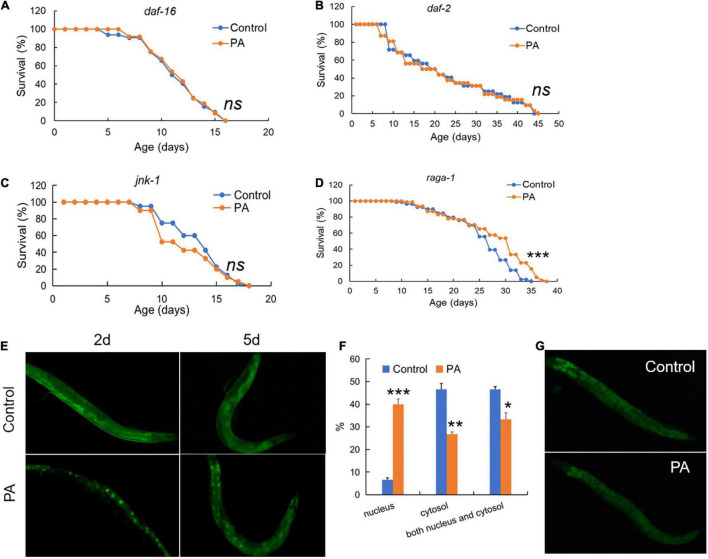
*Pediococcus acidilactici* extends lifespan of *C. elegans* by regulating both *daf-2* and *jnk-1/daf-16* signaling. **(A)** The survival rate of *daf-16* (mgDf50) worms by PA. The control group is *E. coli* OP50 feeding. **(B)** The survival rate of *daf-2* (CB1370) worms, *n* = 80. **(C)** The survival rate of *jnk-1* (gk7) worms by PA. **(D)** The survival rate of *raga-1* (VC222) worms by PA, *n* = 80. ns, non-significant; *p < 0.05; **p < 0.01; ***p < 0.001. **(E)** Feeding daf-16:GFP worms with PA for 2–5 days activates daf-16 expression with the increased nuclear accumulation of daf-16 protein. **(F)** Comparing percentage of daf-16 localization data (cytosolic, nuclear, and both) between *E. coli* control and PA-treated *C. elegans*. **(G)** Feeding daf-2:GFP worms with PA for 5 days represses daf-2 expression.

Common conserved signaling pathways have a profound impact on host prolongevity, such as *daf-2/daf-16* in insulin/IGF-1 signaling and *jnk-1/daf-16* in JNK/MAPK signaling (31). To elucidate the mechanism on lifespan extension mediated by PA, we investigated the fluorescent tracer of *daf-2* and *daf-16* in *C. elegans* and the effects of PA on the lifespan prolongation of *C. elegans* under four mutant backgrounds, such as *daf-2* (CB1370), *daf-16* (GR1307), *raga-1* (VC222), and *jnk-1* (gk7). As a result, deficiency in *daf-2, daf-16*, or *jnk-1* cannot extend the longevity of *C. elegans* by PA (Figures 2A–C), while PA extends the longevity of *C. elegans* with TOR deficiency (Figure 2D), suggesting that the PA-mediated longevity was dependent on insulin/IGF-1 signaling (*daf-2*/*daf-16*) and JNK/MAPK signaling (*jnk-1*/*daf-16*) but not TOR signaling (*raga-1*). In addition, the relocation of *daf-16* to cell nucleus affects the phosphorylation of *daf-16* which is required for life span extension (32). In daf-16:GFP worms, PA increased the nuclear accumulation of *daf-16* at 2 or 5 days probiotic intake (Figure 2E). PA can induce an increase of nuclear daf-16:GFP translocation (*E. coli* OP50: 6.67%; PA: 40%) and a reduction of daf-16:GFP in the cytosolic (*E. coli* OP50: 46.67%; PA: 26.67%) at 5 days (Figure 2F). Additionally, we observed that PA declined the fluorescence intensity of *daf-2* in daf-2:GFP worms (Figure 2G). These results indicated that PA prolonged the longevity *via daf-2*/*daf-16* mediated insulin/IGF-1 signaling, *jnk-1*/*daf-16* mediated JNK/MAPK signaling but not *raga-1* dependent TOR signaling.

### *Pediococcus acidilactici* Reduces Fat Accumulation in *Caenorhabditis elegans*

**FIGURE 3:**
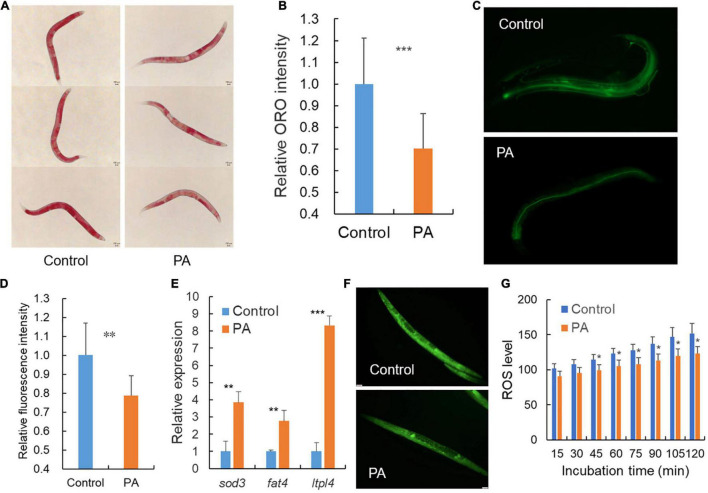
*Pediococcus acidilactici* affects the lipid accumulation of wild-type *C. elegans*. **(A)** Representative images of Oil Red O staining after treatment with *E. coli* OP50 or PA in *C. elegans*, *n* = 45. **(B)** Relative quantification of the Oil Red O staining intensity in **(A)**. **(C)** Representative fluorescence images of fatty acids staining after treatment with *E. coli* OP50 or PA in *C. elegans*, *n* = 20. **(D)** Relative quantification of the green fluorescence intensity in **(C)**. **(E)** Transcript expression of lipid related genes in PA-fed *C. elegans* compared with control *E. coli* OP50. **(F)** Representative images of fluorescence labeled reactive oxygen species (ROS) in *C. elegans*, *n* = 35. **(G)** Relative quantification of the green fluorescence labeled ROS, *p < 0.05; **p < 0.01; ***p < 0.001.

Generally, dye-labeled lipid assays can provide the robust mapping of lipid accumulation especially the intestinal lipid storage in *C. elegans* since the fat is mainly stored in intestinal and epidermal cell (33). Oil Red O is a lysochrome and highly soluble dye in lipids, and can stain triglycerides and lipoproteins in *C. elegans* (34). Compared with the transparent body of *C. elegans*, Oil Red O shows obvious red lipid droplets to allow the qualitative evaluation of lipid distribution (35). Our results revealed a significant decrease in Oil Red O intensity in the worms treated with the probiotic PA compared with control (Figures 3A,B). Recent studies have revealed that regulating lipid metabolism is crucial for longevity in *C. elegans* and the content of triglycerides is a marker reflecting the energy levels (36). PA-treated *C. elegans* reflect a lower fat mass and lower energy, thus contributed to lifespan extension (Figures 3A,B). To explore the cause of lower fat storage, the dietary fat source was detected. As a result, the fluorescence intensity of the BODIPY-labeled fatty acids was significantly decreased in the worms fed with PA compared with the OP50-fed control, suggesting a decrease in fatty acids uptake (Figures 3C,D). In addition, we examined the expression of three key genes involved in the lipocatabolic pathway. As expected, PA-feeding significantly increased the expression of *sod-3*, *fat-4*, and *lipl-4* genes encoding superoxide dismutase, fatty acid desaturase, and triglyceride lipase, respectively (Figure 3E). Excess ROS are genotoxic and can cause oxidative damage within the cell. Further accumulated oxidative stress can induce lipid peroxidation and protein homeostasis interference (37). Here, we found that the fluorescence intensity reflecting ROS levels was significantly reduced by PA-feeding compared with the OP50-fed control (Figures 3F,G), indicating that PA intake can protect host from ROS-induced oxidative damage and lipid peroxidation product accumulation.

### *Pediococcus acidilactici* Promotes the Survival Rate and Age of *Caenorhabditis elegans* by Regulating the Expression of Genes Related to Chloride Ion and Immune Response

**FIGURE 4:**
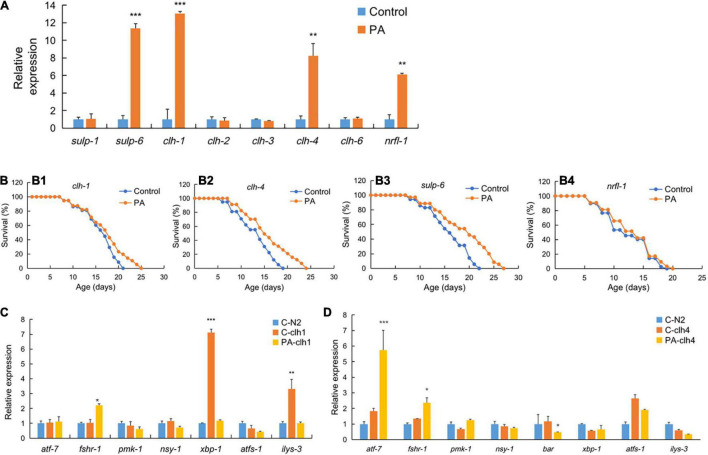
The regulation of genes expression and survival of different mutant worms by PA feeding. **(A)** Expression of chloride ion related genes in PA-fed *C. elegans* compared with control *E. coli* OP50. **(B)** The survival rates of *clh-1* (XA900, **B1**), *clh-4* (RB1139, **B2**), and *sulp-6* (VC1167, **B3**), and *nrfl-1* (VC1795, **B4**) worms by PA feeding, *n* = 80. **(C,D)** The expression of inflammatory or anti-inflammatory genes in PA-fed *clh1* **(C)** or *clh4* **(D)** gene mutant *C. elegans* compared with the mutant control and wild-type N2 feeding *E. coli* OP50, *p < 0.05; **p < 0.01; ***p < 0.001.

To address the novel targets of PA-mediated longevity, we examined the expression of chloride ion related genes in PA- or OP50-fed wild-type *C. elegans*. We found that PA-fed *C. elegans* showed 11-fold increase in *sulp-6*, 13-fold increase in *clh-1*, eight-fold increase in *clh-4*, and six-fold increase in *nrfl-1* transcripts compared with the OP50-fed control (Figure 4A). Most importantly, PA significantly promoted the survival rate and age of *clh-1*, *clh-4*, and *sulp-6* mutant worms (Figures 4B1–B3). In addition, PA had no effect on the longevity of *nrfl-1* mutant worms (Figure 4B4).

The immunoregulatory effect is one of the most important functions of probiotics. Modulating immune response is crucial for the survival of *C. elegans* (38). We examined whether PA regulates the expression of eight genes (*atf-7*, *fshr-1*, *pmk-1*, *nsy-1*, *bar*, *xbp-1*, *atfs-1*, and *ilys-3*) involved in the inflammation of *clh-1* and *clh-4* mutant worms (Figures 4C,D). *C. elegans* is the absence of most part of mammalian innate immune response, e.g., no mobile immune cells and not producing inflammatory cytokines. However, it contains a conserved principal NSY-1/SEK-1/PMK-1 mitogen-activated protein kinase (MAPK) signaling pathway (39). For example, *atf-7* can directly regulate the genes of host defense and the anti-inflammatory PMK-1/ATF-7 signaling can contribute to innate immune response to infection (40). The G protein coupled with receptor *fshr-1*, in parallel to the p38 MAPK pathway, is another important effector triggering innate immune response to ingested pathogens in *C. elegans* (41). Xbox binding protein (*xbp-1*), encoded by an inflammatory gene, can increase the endoplasmic reticulum (ER) folding capacity in the PMK-1-mediated defenses (42). Similarly, the beta-catenin homolog *bar-1* is important for the inflammation of *S. aureus* infection (43). *Ilys-3*, an invertebrate-type lysozyme gene in *C. elegans*, can be upregulated in the intestine with an inflammatory ERK-dependent manner when challenged with pathogens (44). In this study, it is observed that the gene expression of both *xbp-1* and *ilys-3* was relatively lower in PA-fed *clh-1* and OP50-fed wild-type control when compared with the OP50-fed *clh-1* mutant worms, while *fshr-1* expression was significantly increased in PA-fed *clh-1* mutant worms (Figure 4C). Meanwhile, we observed a significant increase in *atf-7* and *fshr-1* expression, but a significant reduction in *bar* expression in PA-fed *clh-4* mutant worms compared with the OP50-fed *clh-4* mutant worms (Figure 4D), implying that PA has immunoregulatory effects on the survival of *C. elegans*.

### Schematic Representation of *Pediococcus acidilactici*-Mediated Lifespan Extension in *Caenorhabditis elegans*

**FIGURE 5:**
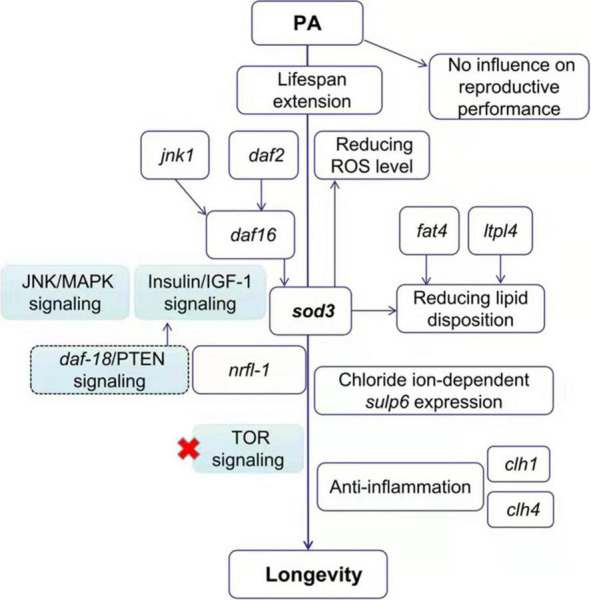
The proposed mechanism of PA-feeding effects on the longevity of *C. elegans*. The PA increases the lifespan of nematodes *via* the Insulin/IGF-1 signaling (*daf-2/daf-16*) and JNK/MAPK signaling (*jnk1/daf-16*) pathway but not Target of Rapamycin (TOR) pathway, inhibiting the fat accumulation, and regulating some of the chloride ion dependent genes.

The probiotic mechanism of PA-feeding effects on *C. elegans* was summarized in Figure 5. In this study, the probiotic PA significantly prolonged the lifespan of *C. elegans* without an influence on reproductive performance. Then, the PA failed to modify the lifespan of *daf-2*, *daf-16* and *jnk-1* mutant worms, implying that the classic Insulin/IGF-1 and JNK/MAPK signaling pathways were implicated in the PA regulated lifespan extension. But this lifespan extension was not dependent on TOR signaling since PA can extend the longevity in the *raga-1* mutant worms. As a downstream of *daf-16*, *sod-3* was required for the antioxidative process to reduce ROS levels. The increased expression levels of *sod-3*, *fat-4*, and *ltpl-4* are contributed to the reducing lipid disposition, and thus promoting longevity. Our findings also pointed to a novel requirement for the *sulp-6*, *nrfl-1, clh-1*, and *clh-4* expression regulating chloride ion related process, emphasizing the possibility that chloride ion is tightly linked to the promoted longevity. The longevity initiated by PA intake was dependent on *nrfl-1* and the *nrfl-1/daf-18* signaling, upstream of Insulin/IGF-1 signaling, might be activated. The *clh-1* or *clh-4* mutations affected the inflammatory gene expression, and PA regulated some anti-inflammatory gene expression and improved longevity. Thus, these data suggest that the PA prolonged lifespan of *C. elegans* by regulating the Insulin/IGF-1 signaling and JNK/MAPK signaling, reducing lipid accumulation, and regulating chloride ion dependent genes, but not dependent on TOR signaling.

## Discussion

Gut beneficial microbes play an important role in the production of age-related metabolites, oxidative stress, and immunoregulation in aging (45). Here, we performed an *in vivo* study to investigate whether probiotics can exhibit anti-aging effect and lead to longevity in the *C. elegans*, and found the extension of lifespan by fermented pickles-origin PA in *C. elegans* (Figures 1–4). Up to date, a lot of probiotics, most of which are LAB strains, have been shown to provide lifespan benefits in *C. elegans* (30). A large-scale strain screening experiment reveals that some LAB strains isolated from Kimchi can prolong lifespan in *C. elegans* (46). Concerning the probiotic safety, feeding the worms with microbes might not affect the reproduction development of the worms. As shown by the results, PA feeding displayed a non-obvious effect on body size, brood size, pharyngeal pumping rate, and reproduction of these worms (Figure 1), though the previous study revealed that body-size reducing was an effect of dietary restriction (47). Thus, the prolonged lifespan effect of PA is independent on dietary restriction. Another TOR pathway evidence further indicates that PA extends lifespan independent on dietary restriction since the reduced TOR signaling is a primary mechanism involved in dietary restriction-mediated extending longevity (48). Some probiotic candidates, such as *Weissella* species and *L. salivarius* can affect the reproduction and locomotor activity which might need some further safety evaluation before application (49, 50).

Several lifespan extension signaling pathways have been identified in *C. elegans*. Among them, the Insulin/IGF-1 signaling plays the critical role in longevity of the *C. elegans* (51). In this study, we demonstrated that the Insulin/IGF-1 pathway, such as the receptor *daf-2* and the downstream transcription factor *daf-16*, was involved in the PA-mediated prolongevity effects. Other studies prove that some probiotic strains can influence aging and longevity *via* the *daf-2*/*daf-16* insulin signaling pathway (52–54). However, the prolongevity regulated by *L. gasseri* SBT2055 in *C. elegans* is independent on the *daf-2*/*daf-16* pathway, and *Bifidobacterium longum* BB68 regulates the lifespan *via daf-16* independent on the *daf-2* pathway, suggesting that the probiotics are strain specific (10, 55). Moreover, the JNK/MAPK signaling is involved in the PA-mediated longevity of *C. elegans*. As an upstream regulator, *jnk-1* interacts with *daf-16* and is considered to be prominently responsible for the immune response and stress (51). *B. longum* BB68 is confirmed to activate the TIR-1/JNK-1/daf-16 signaling and positively regulates longevity (55). Besides, the pro-longevity effects of PA are independent on the *raga-1* pathway.

Reactive oxygen species are known to cause damage to DNA and cumulative genomic damage accelerates aging and influences the health span (56). In this study, the PA showed significant higher ROS-scavenging ability compared with control, and promoted the anti-oxidative *sod-3* gene expression (Figure 3). *B. longum* BB68 is also demonstrated to increase *sod-3* expression which is downstream of the *daf-16* signaling and might contribute to extending the lifespan of *C. elegans* (55). Meanwhile, PA could reduce the absorption of fluorescence-labeled total fatty acids in the intestine, enhance the fatty acid conversion *via fat-4* activation, and increase the lipid hydrolysis *via lipl-4* upregulation, thus reducing fat storage in body as proved by the observation of oil red staining (Figure 3). Consistently, an earlier report finds that the lipid hydrolysis and disposition are modulated by aspirin through *fat-4* and *lipl-4* activation (27). A probiotic *Lactobacillus rhamnosus* CNCM I-3690 is found to inhibit the total fat deposit in *C. elegans via* another fatty acid desaturase gene *fat-7* (54).

To investigate whether PA-feeding activates the chloride channels, we detected the mRNA expression of chloride related genes and performed survival assays on two highly activated gene mutant worms by PA. *clh-1*, *clh-2*, *clh-3*, and *clh-4* are orthologs of human *CLC2* gene which is associated with retina degeneration, constipation, intestinal mucosa repair, epilepsy, and so on (57). Another *sulp-6* is an ortholog of human *SLC26A3*, *SLC26A4*, and *SLC26A5* genes. *SLC26A3* is a key chloride-bicarbonate exchanger protein contributed to infectious diarrhea and inflammatory bowel disease (IBD) (58). *SLC26A4* and *SLC26A5* genes are risk loci for human asthma and hearing loss, respectively (59, 60). Taken together, chloride ion-dependent genes, such as *sulp-6*, *clh-1*, and *clh-4* are associated with the integrity of intestinal mucosa barrier. Consistently, *sulp-6*, *clh-1*, or *clh-4* mutant worms had a decreased lifespan compared with the N2 worms (Figure 4). The significant effect on lifespan of the *sulp-6*, *clh-1*, or *clh-4* mutant worms by PA might be beneficial to intestinal mucosa barrier. More importantly, we identified the *nrfl-1* as the key regulator of longevity initiated by PA intake. *nrfl-1*, which encodes a human ortholog of Na^+^/H^+^ exchanger regulatory factor-1 (NHERF1, also called SLC9A3R1), can interact with chloride CFTR channel and regulate the concentration of Cl^–^/HCO_3_^–^ during the capacitation of spermatozoa (61). There exists a SLC9A3R1/PTEN signaling that NHERF can recruit PTEN to bind PDGFR on membrane, leading to an inhibition of PTEN signaling pathway (62). Another study confirmed that PTEN (a worm ortholog of daf-18) signaling was upstream of insulin/IGF-1 signaling (63). Thus, we propose a mechanism that the *nrfl-1/daf-18* signaling was stimulated by PA feeding, which subsequently activated insulin/IGF-1 signaling, and might play a role in PA-mediated extending lifespan (Figure 5).

The chloride ion plays an important role in the cell-volume regulation, mucus secretion, neuroexcitation development, and so on (64). The increasing evidence reveals that chloride channels and chloride ion dependent genes are involved in various diseases, such as cystic fibrosis, epilepsy, osteopetrosis, myotonia, and hyperekplexia (65). Its beneficial effect regulated by probiotic *Lactobacillus acidophilus* on host stimulation of Cl^–^/OH^–^ exchange activity was first reported in animals (66). Besides, *Bifidobacterium breve* C50 has also been confirmed to promote intestinal homeostasis by controlling chloride ion secretion (67). More importantly, a body tissue chloride ion influx in high fat-fed rats is found in *Lactobacillus casei* intervention group and subsequently this probiotic can prevent colitis by activating chloride ion dependent channel CFTR in mice (68, 69). In this study, the mRNA increase of chloride channel *clh-1* and *clh-4* as well as chloride exchanger *sulp6* which promote the lifespan of *clh-1* or *clh-4* mutant worms might contribute to enhance the intestinal electrolyte absorption and underlie the potential effects of PA (Figure 4). Further, the mRNA increase of chloride-related *nrfl-1* but not prolonging the lifespan of *nrfl-1* mutant worm by PA demonstrated that the PA-mediated lifespan extending was dependent on *nrfl-1*. Besides, we found that PA could increase anti-inflammatory *atf-7* and *fshr-1* mRNA levels in *clh-1* or *clh-4* mutant worms whereas downregulate inflammatory *xbp-1* and *ilys-3* mRNA levels in *clh-1* mutant and *bar* mRNA level in *clh-4* mutant (Figure 4) (70). This might be contributed to the PA-mediated extending lifespan of *clh-1* or *clh-4* mutant worms.

In conclusion, the PA with extending the longevity of *C. elegans* were widely investigated in this study (Figure 5). On one hand, the PA could modulate the *daf-2* expression and nucleus location of *daf-16 via* the Insulin/IGF-1 pathway, and the increased *sod-3* expression was the downstream of *daf-16*. Meanwhile, the PA not only decreased the intestinal fatty acids absorption, but also regulated the lipid hydrolysis and disposition *via fat-4* and *lipl-4* expression, thus reducing ROS accumulation. On the other hand, PA could significantly alter the chloride ion-associated genes mediated inflammatory state and extend the longevity of *C. elegans*. Therefore, it is speculated that the PA might play an important role in the anti-aging activities *via* the modulation of chloride ion-related genes, providing a new perspective to explore the lifespan extending effect. Besides, the study has a vital referential significance for other probiotics screening and provides the theoretical basis for its future application.

## Data Availability Statement

The raw data supporting the conclusions of this article will be made available by the authors, without undue reservation.

## Author Contributions

YZ, XWa, and XWe designed experiments. RH and YZ carried out the experiments, analyzed the experimental results, prepared the original draft, and performed the statistical analysis. WQ, YLe, YLo, and XL finished the validation. YZ, XWa, and XWe reviewed and edited the manuscript. XWa and JL acquired resources. All authors contributed to the article and approved the submitted version.

## Conflict of Interest

YZ, YLe, YLo, XL, JL, XWa, and XWe were employed by Beijing Solidwill Sci-Tech Co., Ltd. The remaining authors declare that the research was conducted in the absence of any commercial or financial relationships that could be construed as a potential conflict of interest.

## Publisher’s Note

All claims expressed in this article are solely those of the authors and do not necessarily represent those of their affiliated organizations, or those of the publisher, the editors and the reviewers. Any product that may be evaluated in this article, or claim that may be made by its manufacturer, is not guaranteed or endorsed by the publisher.
