# Supplementary material for: Pediococcus acidilactici Promotes the Longevity of C. elegans by Regulating the Insulin/IGF-1 and JNK/MAPK Signaling, Fat Accumulation and Chloride Ion
Source: Front Nutr. 2022 Apr 1;9:821685. doi: 10.3389/fnut.2022.821685 (PMC9010657; doi:10.3389/fnut.2022.821685)
Supplement: Supplementary file 1 [file Table_1.DOCX]

**Supplemental Table 1: all the primers used for RT-qPCR analysis in this study**

| Genes | Forward sequence (5'to3') | Reverse sequence (5'to3') |
| --- | --- | --- |
| *clh1* | CAATTCCCCAGACGTTCCTA | AAGATTGCCGTGATGAATCC |
| *clh2* | ACGATAAAATGCTCGCCATC | GACAGAAAATAGCGGCGAAC |
| *clh3* | TACGATCTACAGCCGGGTTC | TGGAATCCTCGTCAATTGGT |
| *clh4* | GTATCGTTTCACCGCGTTTT | ACCCATTCTCAAGTCCATGC |
| *clh6* | CTCGTCGCTGCATTTATTGA | ATACACCCTGCAGTCGTTCC |
| *sulp1* | TCGTGGGCATGACTGTAAAA | CATGGAACAGCTGCGTAAAA |
| *sulp6* | GCTACGGCCTAATCACTGGA | TTCCACAACTGTTCCAACCA |
| *nrfl-1* | GCCATATTTGCCCAGACTTG | CGCGTTCAGCATGAAGATT |
| *sod3* | AGCATCATGCCACCTACGTGA | CACCACCATTGAATTTCAGCG |
| *lipl4* | ATGGCCGAGAAGTTCCTACATCGT | GGTGAATTGGCGACCCAATCGAAA |
| *fat4* | ATTGACTGGCTTTGGGGAGG | CAGTAAGCGCATCCACCAGT |
| *pmk-1* | CCGACTCCACGAGAAGGATA | TCAGCAGCACAAACAGTTCC |
| *nsy-1* | GCTCGTGAAATCACAGATGG | GAGAGCATCATAAGGGATACGG |
| *cdc42* | CTGCTGGACAGGAAGATTACG | CTCGGACATTCTCGAATGAAG |
| *atf-7* | GTTCTTACGAATGCGATGG | CAAGTGGTGAGTGATAGGAG |
| *fshr-1* | AAGCAGAGATGAACCAACA | AAGCATTCCATCAGAAGCA |
| *bar-1* | TACGGCATCTCTGTGTTG | GAATGTCCTGGTCTCTTGTA |
| *xbp-1* | AGAAGGTTAGTGCTGAATGT | GTTGTTGATGGAGGTGGAT |
| *atfs-1* | GGAATGCCACCACAAGAT | CGACGCTGATGATGAAGA |
| *Ilys3* | TGTGCTACTACTTGTGTTGA | CTCTTGATTCCATTCCAGTATC |
